# Correction: Changes in the humoral immunity response in SARS-CoV-2 convalescent patients over 8 months

**DOI:** 10.1038/s41423-022-00915-9

**Published:** 2022-08-30

**Authors:** Pai Peng, Jie Hu, Hai-jun Deng, Bei-zhong Liu, Liang Fang, Kai Wang, Ni Tang, Ai-long Huang

**Affiliations:** 1 Key Laboratory of Molecular Biology for Infectious Diseases (Ministry of Education), Institute for Viral Hepatitis, Department of Infectious Diseases, The Second Affiliated Hospital, Chongqing Medical University, Chongqing, China; 2 Yong-Chuan Hospital, Chongqing Medical University, Chongqing, 402160 China

**Keywords:** Viral infection, Infection

Correction to: *Cellular & Molecular Immunology* 10.1038/s41423-020-00605-4, published online 08 January 2021

The licence information was missing from this article and should have been CC-BY.

The original article has been corrected.

